# Women’s experience of maternal morbidity: a qualitative analysis

**DOI:** 10.1186/s12884-016-0974-0

**Published:** 2016-07-25

**Authors:** S. Meaney, J. E. Lutomski, L. O’ Connor, K. O’ Donoghue, R. A. Greene

**Affiliations:** 1 National Perintal Epidemiology Centre, University College Cork, 5th floor, Cork University Maternity Hospital, Cork, Ireland; 2 Radboud Institute for Health Sciences, Radboud University Medical Center, Nijmegen, Netherlands; 3 Department of Obstetrics and Gynaecology, University College Cork, Cork, Ireland

**Keywords:** Pregnancy, Maternal morbidity, Care, Qualitative

## Abstract

**Background:**

Maternal morbidity refers to pregnancy-related complications, ranging in severity from acute to chronic. In Ireland one in 210 maternities will experience a severe morbidity. Yet, how women internalize their experience of morbidity has gone largely unexplored. This study aimed to explore women’s experiences of maternal morbidity.

**Methods:**

A qualitative semi-structured interview format was utilized. Purposive sampling was used to recruit 14 women with a maternal morbidity before, during or after birth; nine women were diagnosed with one morbidity including hypertensive disorders, haemorrhage, placenta praevia and gestational diabetes whereas five women were diagnosed with two or more morbidities. Thematic analysis was employed as the analytic strategy.

**Results:**

Four superordinate themes were identified: powerlessness, morbidity management, morbidity treatment and socio-behavioural responses to morbidities. Women were accepting of the uncontrollable nature of the adverse outcome experienced. While being treated for trauma, women were satisfied to relinquish their autonomy to ensure the safety of themselves and their babies. However, these events were debilitating. Women’s inability to control their own bodies, as a result of the morbidity, contributed to high levels of frustration and anxiety. Morbidities impacted greatly on women’s quality of life and sometimes these effects persisted for a prolonged period after delivery. Women felt that they were provided very little information on the practicalities of living with their condition; many were uncertain how to manage their morbidities in the home setting.

**Conclusion:**

Healthcare providers should ensure that women who experience a maternal morbidity are fully debriefed and have sufficient information on the morbidity including ongoing care and expectations prior to discharge.

## Background

Pregnancy and childbirth are usually viewed as a positive life event; however, during this time, adverse health issues can arise. The WHO Maternal Mortality Working Group define maternal morbidity as “any health condition attributed to and/or aggravated by pregnancy and childbirth that has a negative impact on the woman’s wellbeing” [1]. Severe maternal morbidity is then defined as “a very ill pregnant or recently delivered woman who would have died had it not been that luck and good care was on her side” [2]. Severe maternal morbidity is considered an indicator of the quality of obstetric care, particularly in developed countries [2]. Globally, there has been an increasing rate of select morbidities, such as gestational diabetes, preeclampsia and postpartum hemorrhage over the past two decades [3–5].

Pregnancy and childbirth often requires substantial psychological adjustment for women [6]; thus, maternal morbidity is not solely limited to physical injury and illness but also includes psychological health. Psychological maternal morbidity is associated with depressive episodes, particularly during the postpartum period [7]. Association with other conditions, such as post-traumatic stress disorder (PTSD) may arise as the result of a birth perceived as traumatic whereby the woman responds with feelings of intense fear and helplessness. [7–9]. It has been reported that up to 6 % of women have severe symptoms of PTSD in the weeks following birth. Moreover, up to 2 % of women may well see these symptoms of PTSD continuing for up to a year following birth [9].

Maternal morbidity can impact a woman both physically and mentally [7] and these negative consequences may transfer to her infant, partner and extended family [7, 10]. These effects can be far reaching including physical, psychological, social and economic consequences [10, 11]. Consequently, maternal morbidities are among the leading causes of disability-adjusted life-years among women aged 15–44 years [10] and increase risk of mortality within 1 year of the morbidity [12]. Moreover, women may not be able to work due to their maternal morbidity and this coupled with high costs for treatment may result in financial strain for the family [10, 11].

Appropriate obstetric care can reduce health risks during pregnancy and childbirth, however, a high prevalence of maternal morbidity may remain unidentified or unreported by health care staff and women [13]. Given the long-lasting effects maternal morbidity can impose on a woman’s psychological and physical health and wellbeing, this study aimed to qualitatively explore the views of women who experienced a maternal morbidity to gain valuable insight into their opinions as they reflected on their care.

## Methods

### Recruitment

Nearly all women in Ireland deliver in a maternity hospital, and thus a purposive sample was recruited from a patient list of those who attended a large tertiary hospital (approximately 8500 births in 2012). The recruitment strategy targeted women who experienced a maternal morbidity either during pregnancy or labour, at birth or shortly after birth. Women were invited to participate by post, and an information letter and opt out form were sent to each woman. Six women returned an opt out form by post and no indication as to why they would not like to participate was given. If there was no indication that the woman would like to opt out of the study, the lead researcher (SM) made contact by telephone to provide more detailed information with regards to the study. If women wished to partake in the study interviews were arranged either at the hospital or at a location convenient for the woman. None of the women were known to the researcher.

### Sample

This study recruited a sample of 14 women, aged 26–38 years, who had experienced a maternal morbidity; eight women with a single morbidity and six women with two or more morbidities (Table 1).

**Table 1:** Sample characteristics

Pseudonym	Patient care status	Parity	Mode of delivery	Morbidity
Sinead	Public	Multiparous	SVD	Gestational Diabetes Tear
Hilda	Private	Primiparous	Elective CS	Placenta previa Transfusion Phlebitis of the cannula site
Therese	Public	Primiparous	SVD	Haemorrhage Retained placenta
Patricia	Private	Primiparous	Elective CS	Hypertension
Catherine	Public	Multiparous	SVD	PET Episiotomy
Siun	Public	Multiparous	VBAC	Postpartum haemorrhage
Margaret	Public	Primiparous	SVD	Postpartum haemorrhage
Naomi	Public	Primiparous	Emergency CS	Anaesthetic problems Postpartum haemorrhage
Abigail	Public	Primiparous	Emergency CS	Placenta previa
Deirdre	Public	Primiparous	Emergency CS	Postpartum haemorrhage Infection
Rachel	Private	Primiparous	SVD	Tear
Loraine	Public	Multiparous	SVD	Septicaemia
Sharon	Private	Primiparous	SVD	Preeclampsia
Laura	Private	Multiparous	SVD	Sickle Cell Crisis

### Data collection and analysis

The lead author (SM), an experienced female qualitative researcher, conducted semi-structured face to face interviews. Interviews took place between 6 and 10 months after the women delivered. Each interview was digitally recorded and contemporaneous notes were taken immediately after each interview. Interview length was 44 min, on average. Each transcript was analysed thematically as it was completed. Thematic analysis is an inductive method which adopts a constant comparative method; therefore, data collection and analysis proceed concurrently until saturation is met. The five stages of analysis, as described by Braun and Clarke [14], first included familiarisation of the data and the identification of the preliminary codes by the researcher. Similar individual codes were then collapsed into categories. The final phases of analysis included the development of themes into a framework which accurately represent the data. The analysis was carried out by SM using Nvivo 10 software (QSR International Pty Ltd., Doncaster, Australia). The reporting of these qualitative results adhere to the COREQ guidelines.

## Results

Analysis of the data suggested four main themes in relation to women’s experiences of maternal morbidity: powerlessness, morbidity treatment, morbidity management and socio-behavioural responses to morbidities. In the following paragraphs, direct quotations from patient interviews are used to illustrate these themes with all names shown as pseudonyms.

### Powerlessness

As women recounted their experiences, they referred not only to the debilitating aspects but the unexpected nature of maternal morbidity. Women felt ill-prepared for what they experienced.

“*It was a bit scary [antepartum haemorrhage: placenta praevia] I guess I wasn’t expecting it as I felt fine…so I rang A&E who said to come in, and once I was in hospital I felt better…you want to be prepared when you go and stay in the hospital for a long time and I wasn’t, it was all spur of the moment at the time*.” (Abigail, Placenta previa)

Women’s inability to control their own bodies as a result of their morbidity contributed to high levels of frustration, anxiety and occasionally fear. These emotions were further exacerbated by poor communication and lack of information from their healthcare providers; women felt they were not clear on what had happened or what to expect to terms of recovery. Women reported that they needed to seek out additional information in an attempt to regain control and to try to make sense of what happened to them. Much of this information was from other women’s personal experiences as well as from various sites on the internet.

*“I hated it. I hated not having any control. You are in the bed surrounded by all these machines that you are strapped up to. You can’t move and, and there is nothing that you can do to make yourself better.” (Lorraine, Septicaemia)*

*“Then the midwife said, ‘Nothing to be worrying about but you seem to be bleeding a little more than what we would consider normal. So we are just going to get the doctor back in.’ And then she came back in and she said that it was that the uterus wasn’t contracting properly and that is why I was losing blood and then she just started pounding on me. So more agony. I just didn’t know exactly what she was doing. I read later on the internet that they can massage it to get it to contract but this wasn’t massaging.” (Margaret, postpartum haemorrhage)*

### Morbidity treatment

In emergency situations, women were more accepting of the uncontrollable nature of their morbidity. While being treated in these situations, women were satisfied to relinquish their autonomy to ensure the safety of themselves and their baby. Women recounted how their memories surrounding the time of treatment were fuzzy; still, they remembered being incoherent at times during the event. Thus, during the treatment process, women were aware of the difficulty faced by clinicians in communicating with them. Given these episodes of incoherence, they expressed that more communication with their partners would be beneficial.

*“Hmm they were doing numbness tests from my head down to my toes to see was I numb or whatever I don’t, husband told me I did sign a consent form, I have no recollection of signing that consent form, I’d tell anybody in that situation don’t ask the mother to sign it cause if you asked me to put my hand on the Bible in the morning, I’d still say I didn’t sign a consent form…I’d no sense of that, I’d no sense of an understanding of what I was going through, bar the fact I wanted my baby to be safe” (Naomi, Anaesthetic problems and postpartum haemorrhage)*

Morbidities greatly impacted women’s quality of life and these effects could persist for a prolonged period of time. Women expressed difficulty adjusting to long term treatment of a maternal morbidity. Women also expressed concern that they were unaware of which medications were available and appropriate for their circumstances.

*“Now I was sore and the midwives kept saying that you only need to take paracetamol and Solpadeine™ and I was like, ‘Come here now love I take more than that for a headache’. I mean what happened to pain is what the patient says it is. In modern medicine I find them stingy* [slang term: insufficient] *with medication; I mean if I had my appendix out I would be on more than just paracetamol and that is in a much less sensitive area.”* (*Rachel, Perineal tear)*

### Morbidity management

Women were dissatisfied with the transition of care from the acute setting, where they received treatment, to general care. They expressed that during treatment either on the labour ward, in the high dependency unit or the intensive care unit the staff were highly responsive to their needs. However, once transferred to the postnatal wards for management of care, they noted a remarkable difference in the level of care provided. Women felt that some general ward staff viewed them as a nuisance and that their requests for assistance unnecessary and/or inconvenient.

*“[Baby] was very unsettled that night and I did have to press the call bell once or twice and I felt the midwife on duty was looking down her nose at me kind of going you’ve only had a Caesarean just get your ass out of bed. Em, thankfully it was a different midwife who came in, I think it was the third time I had pressed the bell … she knew I had come from high dependency and was more than happy to help me with the baby, which put me at ease because I was getting anxious because there was somebody else in the room, I wasn’t sure what to do with the baby, it was taking me so long to get out of bed, I was wrecked tired” (Naomi, Anaesthetic problems and postpartum haemorrhage)*

Women felt that they were provided very little information on the practicalities of their condition; many were uncertain how to manage their morbidities in the home. Women had to reduce or discontinue daily activities resulting in a heavy dependence on family members. This dependence countered the women’s expectations of motherhood as they had not anticipated the morbidity to have such a lasting effect. Upon hospital discharge, the General Practitioner (GP) had a key role in medical monitoring of the morbidity. Women conveyed high levels of trust in their GPs and were satisfied with the care and advice received from their GPs.

*“I remember that morning running my hand under hot water so I would lie so I could say ‘look I can use it’ and it was actually killing me to bend it but just get me out now [from the maternity hospital]…and I had to go to the GP because the blackness kept rising and it was below my elbow and I thought god when is it actually going to stop. And it was black and yellow and grey and every colour under the sun and all my joints were sore…I remember him putting biro* (ink from a pen) *on my elbow and telling me that if it goes over that at the weekend come straight into me on Monday. Now he would deal with a lot of sports injuries … and he said he had only ever seen something like this in a compound fracture” (Hilda, Placenta previa, blood transfusion and phlebitis of the cannula site)*

### Socio-behavioural responses to morbidities

Women expressed concern of the impact of the morbidity on their family. Particular reference was made to husbands/partners who witnessed trauma during delivery.

*“At that stage I was getting a little panicky and started shouting, ‘Is he ok, is he ok?’, and they kinda lifted him up over the screen…then I passed out and after that it is only husbands account of it as I was out of it. He said he was holding the baby and it got kinda like ER…so they said to him to wait outside, so he was born at xx:xx and I didn’t come out till xx:xx [3 h] so as husband says he was outside holding his new born baby with his wife inside bleeding to death as he thought, so he thought he was going to be a single dad.” (Deirdre, postpartum haemorrhage and infection)*

*“So his worst nightmare is coming true all he sees is doctors running to the theatre where I was brought into. He said it could be twenty minutes but it felt like forty before someone came out and said you have a daughter; your wife and your daughter are fine, do you have the baby’s clothes?” (Hilda, Placenta praevia, blood transfusion and phlebitis of the cannula site)*

The safety of the baby was considered the women’s main priority during pregnancy and birth. This prioritisation continued during the postpartum period; at times, caring for their newborn baby resulted in delaying their own recovery. Women stated that it was not until their family had settled into a new routine that they began to reflect back on any complications they experienced themselves.

*“At the time I wasn’t really sure what had gone on or whatever, cause I had to have a blood transfusion as well on the third day. So at the time I didn’t really want to know what had gone on I just knew I had a rough day and needed to focus on getting better. But afterwards when you get home and you think, ‘Hmmm, I don’t think that is as normal as it should be, I don’t think everybody goes through that.’ You are not so sure you haven’t done it before as it’s your first baby, so you start researching it yourself.” (Margaret, postpartum haemorrhage)*

## Conclusion

The findings from this study suggest that the experience of a maternal morbidity has a lasting impact on the women irrespective of the severity of the morbidity. Women especially emphasised the difficulties of managing with morbidities while at home, during both the antenatal and postnatal periods. These difficulties likely stemmed from their dissatisfaction in the amount of information provided to them on how to manage their condition(s) upon discharge. This perceived lack of preparation left the women feeling frustrated and at times inferior both as a woman and as a mother. This was motivated by the inherent belief that they should be able to cope similar other mothers. Similar to the findings from Redshaw et al.*,* women felt they were portrayed as emotional and demanding and therefore felt their requests were considered unreasonable [15]. These feelings were particularly salient in the women who had experienced a perineal tear or a Caesarean delivery; they expressed that healthcare professionals underappreciated the extent of their trauma and its impact on their bodies. These findings mirror those of Baxter et al., who state that there is a need to more fully understand the subjective experience of birth and the threshold at which women themselves define trauma [16]. For instance, women with an uncomplicated spontaneous vaginal delivery may find the birth as traumatising as a woman who had an emergency Caesarean delivery. Importantly, definitions of trauma may not correspond with that of health professionals [16], especially in women who feel ill-prepared for the experience [17]. Thus, understanding the underlying factors affecting how women internalise their birth experience can guide the degree of post-delivery interventions required.

The theme of powerlessness was prominent and comparable to the findings of Redshaw et al. who found that feelings of disempowerment arose when women did not feel that they were being listened to, informed of the situation, or fully understood what happened to them [15]. Feelings of disempowerment endured a number of months after their experience and were compounded by women’s perceived inability to control both the morbidity and their own bodies. These findings support the importance that women place on internal control; such as the ability to maintain control of her own body by adapting behaviours and secondly on external control; such as involvement in decision making with healthcare professionals [9].

This study found that in relation to external control, women were satisfied to relinquish their autonomy during an emergency situation. They fully trusted that healthcare professionals were acting in the best interest of their baby and themselves. The women stated that they would not alter many of the events that occurred but stated how additional communication and clarity in relation to these events would have eased their fears and anxiety. Women were cognisant, however, that healthcare professionals were not always in a position to fully inform women of the course of events during emergency situations. Still, in these situations, improved communication with their partners would be of benefit so accurate information would be conveyed to the women at a later time. This would allow women come to terms with their experience by addressing the potential gaps in their memory which in turn can diminish trauma associated with birth [16].

It is also important to note that poor communication from the medical professionals not only negatively impacted the women but their partners as well. When couples discussed the experience, their partners shared how they often felt disempowered during the event as well. The women stated that their partners often did not know what was happening to their wives or their children since healthcare professionals did not keep them informed during the event. These discussions compounded women’s fears for future pregnancies, and at times, women expressed guilt that they had put their partners in this situation. The women explained that many of these conversations with their partners did not occur for some time after morbidity. The women felt that following delivery, the health and wellbeing of the baby was their priority rather than addressing their own morbidity experience. However, this response may not necessarily be a negative aspect, but rather a useful coping mechanism, helping women recover from their trauma [8]. In this study, it was not until at least 1 month after discharge that the women felt they began to enquire in order to come to terms with what exactly had happened to them. Still, in light of this transitional period, debriefing sessions should take place no earlier than a month after delivery [6, 16, 18]. Such sessions should target both mothers and fathers to address feelings of powerlessness, fear and guilt. Debriefing sessions allow for women to have a greater understanding of their experience by allowing them to recount their experience and receive acknowledgment that their experiences were genuinely difficult and of no fault of their own [7, 16].

### Strengths and limitations

The findings of qualitative studies are context specific and therefore are not generalisable in the same manner as quantitative findings. However, qualitative analysis allows for in-depth exploration of people’s opinions, beliefs and perceptions in order to shed light on a given phenomenon. This study aimed to provide a detailed account of the experiences of women and therefore it was felt that a qualitative methodology was most appropriate.

Throughout the interview process, the women identified how their experience had considerable impact on their husbands/partners as well as extended family members. Thus, family member experiences are solely described from the viewpoint of the women. Nonetheless, this limitation is not inherent to this study; women often act as gatekeepers for men (and other family members) in maternity-related research. Still, the information on the perspectives of the family members should not be underestimated, since the women’s perception of their family’s struggles also act a source of stress and anxiety for women.

## Conclusion

Lack of information due to poor communication was the strongest element which arose from these women’s narratives. Findings from this study illustrate the importance of consultation between health professionals and women before discharge to ensure they are debriefed on the traumatic event as well as provided detailed information on coping with morbidities in the home setting. Counselling support should be made available to both mothers and fathers after a severe morbidity event. Ideally, such support would be available several months postpartum, after women have begun to process the event and recognise the magnitude of the event on their everyday life.
